# Chicago Medical Society, July 20, 1874

**Published:** 1874-08-01

**Authors:** Will. T. Montgomery


### CHICAGO MEDICAL SOCIETY.

### Regular Semi-Monthly Meeting, July 20, 1874.

*Reported by Will. T. Montgomery, M.D.*

THE Society met as usual in the parlor of the Galt House, the President in the chair. In the absence of the Secretary, Dr. Graham was elected Secretary *pro tem.*

Dr. A. B. Strong read a paper on the Pulsation of the Fœtal Heart, Dr. D. A. K. Steele presenting a supplementary report. On motion, it was agreed that both papers be published in full in the report of the Society.

Dr. C. M. Fitch said he had made eleven examinations with a view to determine sex, and that he had predicted correctly in nine cases. When the pulsations were over 140 per minute, he predicted the birth of a female child; when under 130, that of a male infant. A rate between these numbers he concluded insufficient as a basis upon which to form a conclusion. He thought a stethoscope with a metallic bell preferable in these examinations. Dr. Paoli could not see any practical importance in determining the sex of the fœtus. He was not aware that the sex made any difference with regard to preparing clothing, etc. He could see how it might be of importance to crowned heads to know the sex before birth, in that they might be prepared to celebrate the birth of prince or princess, as the case might be. Dr. Bridge said that while the determining of sex may not be of practical importance, the facts which may be gained in reference to the health and presentation of the child and the condition of the placenta are of much importance.

Dr. T. D. Fitch—We may very well say that these investigations are of no practical interest, but they are of much scientific interest. He did not think it practically important to determine before birth the condition of the child or placenta, as the physician should be prepared for any emergency that might arise.

Dr. Seely—It may not be necessary to determine the sex before birth, but if we are able to do this we may thus gain the confidence of our patient, which is often an acquisition of great importance.

Dr. Montgomery had made examinations in a number of cases with reference to determining sex, and was generally correct in predicting the birth of a female child when the pulsations were over 130, and that of a male child when they were under 130.

Dr. Strong said that in the first eight cases which he had examined he was correct in predicting the sex, and he thought a greater number of cases were required if we wished to aim at conclusions of value. He referred to a case of contracted pelvis, reported by Dr. Wilson, in which the determining of sex in utero was of practical importance. Dr. Steele thought the determining of sex in utero might be of value in a medico-legal point of view.

Dr. Van Buren wished to know if there is any truth in the theory that gestations of mothers carrying male children are longer than those carrying female? He also wished to know what effect protracted labors have upon the fœtal pulsations? Dr. C. M. Fitch had seen a child delivered after a protracted labor of forty hours. The child proved to be idiotic and he thought this was the result of the long continued pressure. Dr. Stillians had seen a similar case, and thought the injurious effect of continued pressure was an argument against the use of ergot. Dr. T. D. Fitch said, in reference to the length of gestations, he had observed that those of mothers carrying male children were longest, and if gestation was prolonged beyond term he was generally correct in predicting the birth of a boy. Dr. Quine did not know that much reliance could be placed on the duration of gestation. He had made a number of observations in reference to determining sex by auscultation, and had usually been correct. The position could not be so readily determined by this as by external manipulations. He thought inflammation of the placenta could be ascertained by repeated auscultations.

Dr. Bartlett—An exceedingly slow pulse, as low as seventy or eighty, is very unfavorable to the child, and when it is observed, delivery should be effected as soon as possible. He had been summoned within the last week to attend a lady five miles distant, and when he arrived at the house he learned that she merely desired information as to the sex of her future child.

Dr. Foster did not believe the duration of gestation was a decided indication as to sex. He had recently attended a lady on the 285th day of gestation and delivered her of a female infant.

Dr. Bridge gave an explanation of the marked influence the contractions of the uterus exert over the fœtal pulsations. He thought the great diminution in the number of pulsations at the moment of greatest contraction was due to the interruption of the circulation, so that the blood in the placenta and fœtus became noxious from nonæration. The Society then adjourned.

